# Supplementary material for: MetaRibo-Seq measures translation in microbiomes
Source: Nat Commun. 2020 Jun 29;11:3268. doi: 10.1038/s41467-020-17081-z (PMC7324362; doi:10.1038/s41467-020-17081-z)
Supplement: Supplementary file 10 — Supplementary Data 7 [file 41467_2020_17081_MOESM10_ESM.zip › File2/Confidence_High_Taxonomy/275493_out.krona.html]

Javascript must be enabled to view this page.

members
magnitude
magnitudeUnassigned
count
unassigned
taxon
rank

275493\_out

237

2
237
superkingdom

976
phylum
237

200643
237
class

237
5
order
171549

SRS014948\_contig\_number\_16629SRS017191\_contig\_number\_contig-100\_884.111510SRS017521\_contig\_number\_5912SRS024132\_contig\_number\_48239SRS143372\_contig\_number\_contig-100\_1513.86196

family
211
2005525

211
39
genus

SRS014313\_contig\_number\_641SRS016954\_contig\_number\_5758SRS019397\_contig\_number\_11725SRS019601\_contig\_number\_22380SRS019685\_contig\_number\_37700SRS019910\_contig\_number\_25880SRS023583\_contig\_number\_16828SRS023715\_contig\_number\_18172SRS023829\_contig\_number\_contig-100\_248.12012SRS024625\_contig\_number\_2587SRS043667\_contig\_number\_2746SRS047014\_contig\_number\_35353SRS048870\_contig\_number\_20231SRS049712\_contig\_number\_17257SRS051031\_contig\_number\_27348SRS051610\_contig\_number\_9249SRS056519\_contig\_number\_18036SRS056537\_contig\_number\_4721SRS064557\_contig\_number\_22733SRS077294\_contig\_number\_14183SRS077392\_contig\_number\_contig-100\_100.229163SRS077454\_contig\_number\_contig-100\_532.116510SRS078176\_contig\_number\_18093SRS1041133\_contig\_number\_20467SRS1041134\_contig\_number\_8996SRS1041136\_contig\_number\_9966SRS1041137\_contig\_number\_29492SRS1041144\_contig\_number\_contig-100\_1246.34848SRS1041147\_contig\_number\_1829SRS105082\_contig\_number\_18237SRS1054716\_contig\_number\_552SRS1055069\_contig\_number\_11131SRS1055095\_contig\_number\_contig-100\_1094.1095SRS142599\_contig\_number\_28496SRS143085\_contig\_number\_2312SRS144297\_contig\_number\_14644SRS148721\_contig\_number\_46760SRS149181\_contig\_number\_18494SRS893341\_contig\_number\_11456
375288

species
2
563193

SRS075078\_contig\_number\_16370SRS142890\_contig\_number\_22476

46503

SRS014235\_contig\_number\_27188SRS019968\_contig\_number\_10745SRS045244\_contig\_number\_770SRS045645\_contig\_number\_32534SRS076976\_contig\_number\_13495SRS103987\_contig\_number\_19160SRS1055076\_contig\_number\_contig-100\_171.89460SRS140492\_contig\_number\_32176
species
8

species
2
2293116

SRS019030\_contig\_number\_2677SRS078665\_contig\_number\_9669

1
species

SRS023971\_contig\_number\_641
469591


SRS011084\_contig\_number\_27864SRS011239\_contig\_number\_1501SRS012273\_contig\_number\_44690SRS012969\_contig\_number\_37344SRS013158\_contig\_number\_524SRS013951\_contig\_number\_29623SRS014459\_contig\_number\_9315SRS015065\_contig\_number\_2570SRS015217\_contig\_number\_18024SRS015782\_contig\_number\_43661SRS015794\_contig\_number\_4992SRS015890\_contig\_number\_17114SRS015960\_contig\_number\_11718SRS016056\_contig\_number\_5532SRS016095\_contig\_number\_21723SRS016267\_contig\_number\_8890SRS016335\_contig\_number\_39608SRS016989\_contig\_number\_15456SRS017103\_contig\_number\_36193SRS017307\_contig\_number\_26868SRS017433\_contig\_number\_14911SRS017701\_contig\_number\_19166SRS018313\_contig\_number\_11712SRS018351\_contig\_number\_4681SRS018541\_contig\_number\_2563SRS018575\_contig\_number\_6145SRS018623\_contig\_number\_1162SRS018656\_contig\_number\_9988SRS018836\_contig\_number\_25875SRS018888\_contig\_number\_673SRS018984\_contig\_number\_4603SRS019178\_contig\_number\_524SRS019267\_contig\_number\_550SRS019496\_contig\_number\_14959SRS019787\_contig\_number\_688SRS020328\_contig\_number\_30133SRS020394\_contig\_number\_14292SRS021219\_contig\_number\_12216SRS021948\_contig\_number\_23153SRS022071\_contig\_number\_32642SRS022713\_contig\_number\_7779SRS023526\_contig\_number\_34119SRS024331\_contig\_number\_32475SRS024388\_contig\_number\_1045SRS043701\_contig\_number\_655SRS045195\_contig\_number\_981SRS045826\_contig\_number\_18999SRS046712\_contig\_number\_145SRS047044\_contig\_number\_12197SRS049900\_contig\_number\_14119SRS050026\_contig\_number\_10928SRS050752\_contig\_number\_27409SRS050925\_contig\_number\_33993SRS051882\_contig\_number\_13832SRS052027\_contig\_number\_2602SRS052078\_contig\_number\_572SRS052697\_contig\_number\_945SRS053214\_contig\_number\_15472SRS053398\_contig\_number\_19908SRS053573\_contig\_number\_39SRS053649\_contig\_number\_17405SRS054590\_contig\_number\_592SRS056259\_contig\_number\_28191SRS056273\_contig\_number\_29392SRS057478\_contig\_number\_2444SRS058145\_contig\_number\_3805SRS058770\_contig\_number\_25792SRS062701\_contig\_number\_2252SRS063040\_contig\_number\_30500SRS064645\_contig\_number\_497SRS064973\_contig\_number\_2725SRS074964\_contig\_number\_contig-100\_49.57693SRS075398\_contig\_number\_511SRS075984\_contig\_number\_20566SRS076804\_contig\_number\_17256SRS077127\_contig\_number\_15125SRS077194\_contig\_number\_22644SRS077849\_contig\_number\_31380SRS078419\_contig\_number\_33281SRS097920\_contig\_number\_14950SRS098571\_contig\_number\_13005SRS100021\_contig\_number\_10734SRS104036\_contig\_number\_7524SRS1041036\_contig\_number\_567SRS1041039\_contig\_number\_23585SRS1041091\_contig\_number\_20242SRS1041095\_contig\_number\_3010SRS1041129\_contig\_number\_11134SRS1041132\_contig\_number\_949SRS1041138\_contig\_number\_1495SRS1041142\_contig\_number\_7140SRS1041145\_contig\_number\_14736SRS104327\_contig\_number\_6480SRS104693\_contig\_number\_9063SRS1055038\_contig\_number\_5175SRS142503\_contig\_number\_46408SRS142542\_contig\_number\_16114SRS143148\_contig\_number\_12635SRS143342\_contig\_number\_732SRS144362\_contig\_number\_13814SRS144603\_contig\_number\_1458SRS145497\_contig\_number\_13182SRS146764\_contig\_number\_29725SRS147272\_contig\_number\_323SRS147377\_contig\_number\_5007SRS147614\_contig\_number\_1830SRS148196\_contig\_number\_22091SRS148253\_contig\_number\_1584SRS148511\_contig\_number\_15899SRS148784\_contig\_number\_17225SRS148817\_contig\_number\_12064SRS148874\_contig\_number\_2628SRS149244\_contig\_number\_9252SRS893187\_contig\_number\_18091SRS893231\_contig\_number\_244SRS893253\_contig\_number\_680SRS893259\_contig\_number\_463SRS893285\_contig\_number\_2116SRS893288\_contig\_number\_126SRS893342\_contig\_number\_7654SRS893382\_contig\_number\_6960SRS893383\_contig\_number\_26868
823
122
species


SRS014287\_contig\_number\_3907
2293115
1
species

1
species

SRS1055049\_contig\_number\_9854
1897013

1
species

SRS015431\_contig\_number\_41654
387661

21
species

SRS011134\_contig\_number\_35791SRS015579\_contig\_number\_26079SRS015663\_contig\_number\_35733SRS019068\_contig\_number\_109710SRS020622\_contig\_number\_3385SRS022609\_contig\_number\_42951SRS046369\_contig\_number\_10716SRS048060\_contig\_number\_10666SRS058723\_contig\_number\_8031SRS063370\_contig\_number\_6573SRS075773\_contig\_number\_contig-100\_214.234328SRS075878\_contig\_number\_17997SRS078242\_contig\_number\_25000SRS098061\_contig\_number\_4098SRS098655\_contig\_number\_9254SRS098717\_contig\_number\_14118SRS1041090\_contig\_number\_900SRS143523\_contig\_number\_contig-100\_127.137878SRS147271\_contig\_number\_25603SRS893252\_contig\_number\_13632SRS971275\_contig\_number\_30906
574930


SRS020233\_contig\_number\_58266SRS045004\_contig\_number\_28248SRS045713\_contig\_number\_20829SRS049773\_contig\_number\_29033SRS065504\_contig\_number\_13444SRS1041092\_contig\_number\_9016SRS1041141\_contig\_number\_2157SRS143876\_contig\_number\_31011SRS144183\_contig\_number\_32431
2293117
9
species


SRS015264\_contig\_number\_10367SRS050299\_contig\_number\_12801SRS077086\_contig\_number\_2337SRS971276\_contig\_number\_10007
1947048
4
species

171551
family
5

836
genus
5

species
5
658663

SRS015190\_contig\_number\_16439SRS043411\_contig\_number\_11730SRS050422\_contig\_number\_33587SRS077589\_contig\_number\_7300SRS147346\_contig\_number\_16268

1853231
1
family

283168
genus
1

species
1
28118

SRS077730\_contig\_number\_18426

815
family
11

816
11
genus

47678

SRS104975\_contig\_number\_19676
species
1

species
4
821

SRS014412\_contig\_number\_9212SRS077335\_contig\_number\_526SRS097958\_contig\_number\_11769SRS146813\_contig\_number\_15606


SRS050941\_contig\_number\_14115
46506
1
species


SRS893256\_contig\_number\_547
28116
1
species

species
2
246787

SRS013098\_contig\_number\_20535SRS065397\_contig\_number\_13160

1841856

SRS013800\_contig\_number\_24819
species
1

species
1
357276

SRS142712\_contig\_number\_1307

family
2
171550

239759
2
genus

1
species

SRS019445\_contig\_number\_30479
328814

1
species

SRS104311\_contig\_number\_28761
1872444

2
family
171552

838
genus
2


SRS144506\_contig\_number\_52651
165179
1
species

species
1
28125

SRS043768\_contig\_number\_6539
